# Generation of Ince–Gaussian Beams Using Azocarbazole Polymer CGH

**DOI:** 10.3390/jimaging8050144

**Published:** 2022-05-21

**Authors:** Sumit Kumar Singh, Honoka Haginaka, Boaz Jessie Jackin, Kenji Kinashi, Naoto Tsutsumi, Wataru Sakai

**Affiliations:** 1 Doctor’s Program of Materials Chemistry, Graduate School of Science and Technology, Kyoto Institute of Technology, Kyoto 606-8585, Japan; getsumitkumarlive@gmail.com; 2 Bachelor’s Program of Materials Chemistry, Graduate School of Science and Technology, Kyoto Institute of Technology, Kyoto 606-8585, Japan; hono0319.h@icloud.com; 3 Materials Innovation Laboratory, Kyoto Institute of Technology, Kyoto 606-8585, Japan; 4 Faculty of Materials Science and Engineering, Kyoto Institute of Technology, Kyoto 606-8585, Japan; tsutsumi@kit.ac.jp (N.T.); wsakai@kit.ac.jp (W.S.)

**Keywords:** Ince–Gaussian beam, computer-generated holography, azocarbazole polymer, digital hologram printing, optical communication, Python source code

## Abstract

Ince–Gaussian beams, defined as a solution to a wave equation in elliptical coordinates, have shown great advantages in applications such as optical communication, optical trapping and optical computation. However, to ingress these applications, a compact and scalable method for generating these beams is required. Here, we present a simple method that satisfies the above requirement, and is capable of generating arbitrary Ince–Gaussian beams and their superposed states through a computer-generated hologram of size 1 mm^2^, fabricated on an azocarbazole polymer film. Other structural beams that can be derived from the Ince–Gaussian beam were also successfully generated by changing the elliptical parameters of the Ince–Gaussian beam. The orthogonality relations between different Ince–Gaussian modes were investigated in order to verify applicability in an optical communication regime. The complete python source code for computing the Ince–Gaussian beams and their holograms are also provided.

## 1. Introduction

The solution to the paraxial wave equation in elliptical coordinates can be expressed in terms of an Ince polynomial. The electromagnetic wave generated in accordance with this solution is known as an Ince–Gaussian (IG) beam. These beams form a complete orthogonal basis in Hilbert space and can be converted into other structural beams such as a Laguerre–Gaussian (LG) beam (in cylindrical coordinates) and Hermite–Gaussian (HG) beam (in cartesian coordinates), by varying their elliptical parameters [1]. Hence, IG beams are considered as one of the most generalized beams. IG beams have advantages over LG and HG beams due to their higher spatial degree of freedom and these beams can also be used as a fundamental mode which can generate other complex vector optical fields [2]. IG beams find application in various fields, such as quantum information [3], optical communication [4], optical storage [5], biological medicine [6], optical trapping [7] and non-linear optics [8]. In order to harness the benefits of IG beams, a simple and efficient method to generate these beams is important. A variety of different tools and methods for the generation of IG beams have been reported. An IG beam was first observed by Schwarz et al. [9] and Bandres et al. [10] in a stable resonator. Later, Otsuka et al. [11] reported generation by breaking the symmetry of a cavity in a solid state laser under a tightly focused pump beam. Ren et al. [12] reported the arbitrary generation of Ince–Gaussian beams using a digital micromirror device (DMD), which is an array of 1024 × 768 micromirrors with a switching frequency of 5.2 kHz. Aguirre et al. [13] used kinoform phase elements for the generation of IG and HG beams. The generation of a helical Ince–Gaussian beam was reported by Bentley et al. [14], who used a complex amplitude and phase mask encoded on a liquid crystal display. Wang et al. [15] demonstrated Ince–Gaussian-beam array generation by using a computer-generated hologram (CGH) and spatial light modulator (SLM). A second harmonic Ince–Gaussian beam generation was reported by Wang et al. [16] by employing a binary non-linear CGH. Ohtomo et al. [17] demonstrated a method to generate vortex, HG and IG beams, and also the conversion of one structural beam to another using an astigmatism mode converter (AMC).

All the existing methods, tools and techniques for generating Ince–Gaussian and other similar structural beams require a bulky system assembled on an optical bench or a complex fabrication method (e.g., metamaterials) [18]. We have recently shown that compact vortex beam generators can be realized by using azocarbazole CGH [19], which is simple and compact. In this paper, we further extend the method to incorporate the more general IG beams and their generation. The method combines a digital hologram printing (DHP) technique, CGH calculation and photoisomeric polymer fabrication, to generate arbitrary IG beams and their superposition states. We printed a CGH of an IG beam on an azocarbazole polymer film of size 1 mm${}^{2}$, which is simple and small compared to the existing popular devices such as SLM. The printed hologram can be used to reconstruct an IG beam and its superposition states without any complex optical setup. In summary, the following advantages in the generation of IG beams can be expected when comparing to the conventional methods that use SLM/DMD:

- The two methods reported for the generation of arbitrary IG beams are LCoS-SLM and DMD, which have a pixel pitch of 3 microns. This results in a small diffraction angle and they are always required to have a 4f Fourier-filtering setup to separate out the zero-order beam from the diffracted beam. The digitally printed hologram on azopolymers has a pixel pitch of 0.8 microns and does not require any optics in the reconstruction setup. This provides a significant reduction in system size footprint compared to SLM/DMD.
- The printed holograms are just 1 mm in size and can be used in an integrated optical device (after printing), which cannot be achieved using SLM/DMD.
- Digital hologram printing allows to print large-sized holograms (25 cm) at a pixel pitch of 0.8 microns, thereby offering a huge space–bandwidth product. This can be beneficial in multiplexing 1000’s of beam modes, which is a crucial requirement in information processing and communication applications. To do the same using SLM/DMD requires spatially tiling SLMs which significantly increases the cost and size footprint.

It is noted that azocarbazole polymer also differs from the other popular holographic polymer called a photorefractive polymer. A photorefractive polymer is a real-time read/write material, where both the writing beams and reading beams must be present all the time, which increases system complexity. Since the purpose of the research reported in this paper is to develop a compact system without any optics in the readout process, photorefractive polymers were not considered. Moreover, the photoinduced birefringence exhibited by the azo polymers assists in obtaining a high diffraction efficiency of 30%, which is indeed helpful. Complications with respect to polarization states can be avoided, if the light beams present during recording have a reasonably pure polarization state.

In order to generate the CGH of an IG beam, it is first required to compute the complex amplitudes (of the beam cross-section) corresponding to each mode number at a particular propagation distance. Earlier reports used proprietary software such as MATLAB [20] and Mathematica [21] to realize these computations. Here, we implement the same using Python3 [22], which is open source and freely available. We also provide the Python source, in Supplementary Materials, that can reproduce all the results presented in the paper. Intensity and phase profiles corresponding to different IG beams and their derivatives (LG and HG) were successfully generated and verified experimentally. The superposition states of different IG beams that correspond to different elliptical parameters have also been generated and their orthogonality relations were investigated.

## 2. Theory and Methods

### 2.1. Theory

#### 2.1.1. Ince–Gaussian Beam

In this section, we will discuss the theoretical foundation of an IG beam and some of its characteristics. The mathematical origin of this beam can be traced back to the Helmholtz equation (also known as the wave equation), which is defined as follows,

(1) $$\nabla^{2}U+k^{2}U=0$$

where *k* is a wave number. Now, for the beam propagating in the *z* direction, the solution of Equation (1) can be described as

(2) $$U\left(x,y,z\right)=U_{0}\left(x,y,z\right)e^{ikz}$$

Combining Equations (1) and (2) under slow-varying approximation of wave function ($U_{0}$), the Helmholtz equation changes to

(3) $$\frac{\partial^{2}U_{0}}{\partial x^{2}}+\frac{\partial^{2}U_{0}}{\partial y^{2}}+2ik\frac{\partial U_{0}}{\partial z}=0$$

**Figure 1:**
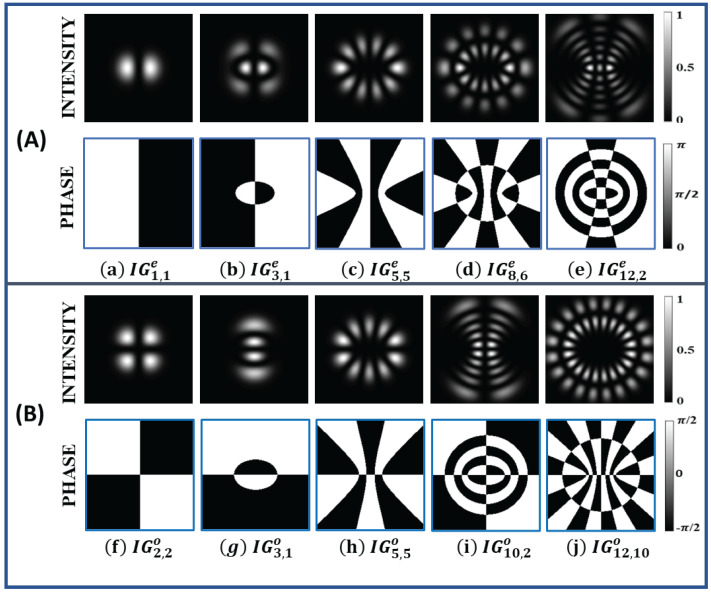
(**A**) Intensity (first row) and phase (second row) profile of even Ince−Gaussian beams (**a**) $IG_{1,1}^{e}$, (**b**) $IG_{3,1}^{e}$, (**c**) $IG_{5,5}^{e}$, (**d**) $IG_{8,6}^{e}$, (**e**) $IG_{12,2}^{e}$ for ϵ=2. (**B**) Intensity (first row) and phase (second row) profile of odd Ince−Gaussian beams (**f**) $IG_{2,2}^{o}$, (**g**) $IG_{3,1}^{o}$, (**h**) $IG_{5,5}^{o}$, (**i**) $IG_{10,2}^{o}$, (**j**) $IG_{12,10}^{o}$ for ϵ=2.

The lowest order solution to Equation (3) yields a Gaussian beam defined as

(4) $$U\left(r\right)=A\left(\frac{W_{0}}{W(z)}\right)e^{-\frac{r^{2}}{W^{2}\left(z\right)}}e^{\left(i(kz+\frac{kr^{2}}{2R(z)}-\psi \left(z\right))\right)}$$

where *A* is normalization constant, $r=\sqrt{\left(x^{2}+y^{2}\right)}$, *W*(*z*) is the spot size of the beam $\left(W\left(z\right)=W_{0}\sqrt{1+{\left(\frac{Z}{Z_{R}}\right)}^{2}}\right)$, such as $Z_{R}$ is the Rayleigh range, and $W_{0}$ is beam width at *z* = 0, *R*(*z*) is radius of curvature $\left(R\left(z\right)=Z+\frac{Z_{R}^{2}}{Z}\right)$ and ψ(z) is the Gouy phase (ψ(z)=arctan $\left(\frac{Z}{Z_{R}}\right))$. The higher order solution to Equation (1) in elliptical coordinate system under paraxial approximation is given by

(5) $$IG_{p,m}^{o}\left(r,\epsilon \right)=S\left(\frac{W_{0}}{W(z)}\right)S_{p}^{m}\left(i\zeta ,\epsilon \right)S_{p}^{m}\left(\eta ,\epsilon \right)e^{-\frac{r^{2}}{W^{2}\left(z\right)}}e^{\left(i(kz+\frac{kr^{2}}{2R(z)}-\left(p+1\right)\psi \left(z\right))\right)}$$

(6) $$IG_{p,m}^{e}\left(r,\epsilon \right)=C\left(\frac{W_{0}}{W(z)}\right)C_{p}^{m}\left(i\zeta ,\epsilon \right)C_{p}^{m}\left(\eta ,\epsilon \right)e^{-\frac{r^{2}}{W^{2}\left(z\right)}}e^{\left(i(kz+\frac{kr^{2}}{2R(z)}-\left(p+1\right)\psi \left(z\right))\right)}$$

where *S* and *C* are the normalization constants, indices *o* and *e* denote the odd and even Ince–Gaussian polynomial and $S_{p,m}$ and $C_{p,m}$ are odd and even Ince polynomials. These polynomials are defined with order *p* and degree *m*, such that *p* and *m* must have the same parity (${\left(-1\right)}^{p-m}=1$) and obey the condition 0≤m≤p for odd Ince polynomials and 1≤m≤p for even Ince polynomials. In above equations, ζ and η denote the radial and angular elliptical coordinates, respectively, which are defined as *x* = fcoshζcosη and *y* = fsinhζsinη, such that ζ∈[0,∞), η∈[0,2π) and ϵ is elliptical parameter defined as $\epsilon =\frac{2f^{2}}{W^{2}}$, where f is the semifocal separation. The beam thus generated and corresponding to the Ince–Gaussian polynomial is known as the Ince–Gaussian beam. The intensity and phase profile of even and odd Ince–Gaussian beams are shown in Figure 1.

The normalization constant of IG beam can be obtained using the orthogonality relation given as

(7) $$\int \int \left(IG_{p,m}^{\sigma }\left(\zeta ,\eta \right)\right)\times \left(IG_{p^{\prime },m^{\prime }}^{\sigma^{\prime }}\left(\zeta ,\eta \right)\right)ds=\delta_{\sigma \sigma^{\prime }}\delta_{pp^{\prime }}\delta_{mm^{\prime }}$$

The linear combination of odd and even Ince–Gaussian beams form a helical Ince–Gaussian beam, which is defined as

(8) $$IG_{p,m}^{h}\left(\zeta ,\eta \right)=IG_{p,m}^{e}\left(\zeta ,\eta \right)+iIG_{p,m}^{o}\left(\zeta ,\eta \right)$$

**Figure 2:**
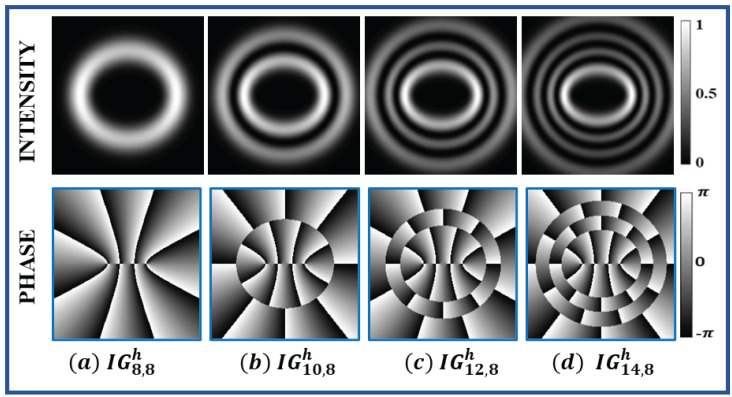
Intensity (first row) and phase profile (second row) profile of helical Ince−Gaussian beams (**a**) $IG_{8,8}^{h}$, (**b**) $IG_{10,8}^{h}$, (**c**) $IG_{12,8}^{h}$, (**d**) $IG_{14,8}^{h}$ for ϵ=2.

Some of the helical Ince–Gaussian beams can form an elliptical vortex followed by elliptical rings. The total number of rings of these kinds of helical Ince–Gaussian beams, ( $IG_{p,m}^{h}\left(\zeta ,\eta \right)$), are equal to $1+\frac{p-m}{2}$. The intensity and phase profile of these particular kinds of helical Ince–Gaussian beams are shown in Figure 2.

**Figure 3:**
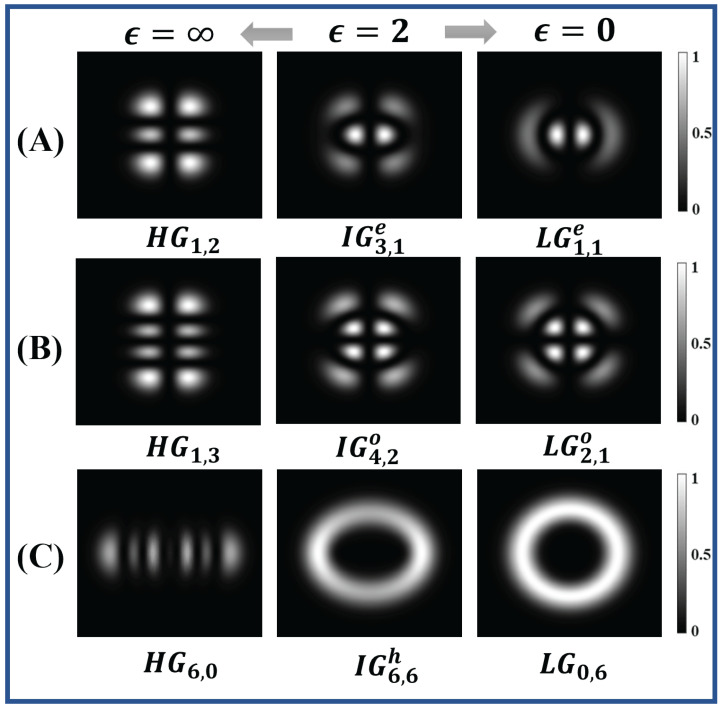
Transformation of Ince–Gaussian beam (center) into Hermite–Gaussian beam (left) and Laguerre–Gaussian beam (right) at different elliptical parameter (ϵ) values. (**A**) Conversion of $IG_{3,1}^{e}$ for ϵ=2 into $HG_{1,2}$ for ϵ=∞ and $LG_{1,1}$ for ϵ=0. (**B**) Conversion of $IG_{4,2}^{o}$ for ϵ=2 into $HG_{1,3}$ for ϵ=∞ and $LG_{2,1}$ for ϵ=0. (**C**) Conversion of $IG_{6,6}^{h}$ for ϵ=2 into $HG_{6,0}$ for ϵ=∞ and $LG_{0,6}$ for ϵ=0.

The IG beam can be transformed into other structural beams, such as HG and LG, by varying its elliptical parameter (ϵ). When ϵ = 0, the $IG_{p,m}^{\sigma }$ beam changes to LG ($LG_{n,l}^{\sigma }$) beam, such as l=m and $n=\frac{p-m}{2}$ and for ϵ=∞ the $IG_{p,m}^{\sigma }$ beam changes to HG ($HG_{n_{x},n_{y}}^{\sigma }$) beam, such as $n_{x}$ = m−1 and $n_{y}$ = p−m+1 for σ = odd and $n_{x}$ = *m* and $n_{y}$ = p−m for σ = even. The transformations of IG beam to LG and HG beam for different elliptical parameter values are shown in Figure 3. All these structural beams form a complete set in Hilbert space, so any one beam (e.g., LG, HG, IG) can be written in terms of other beams, as shown in Equation (9).

(9) $$IG_{p,m}^{\sigma }\left(\zeta ,\eta \right)=\sum_{n,l}A_{n,l}\left(LG_{l,m}^{\sigma }\left(r,\phi \right)\right)=\sum_{a,b}B_{a,b}\left(HG_{n_{x},n_{y}}\left(x,y\right)\right)$$

where $A_{n,l}$ and $B_{a,b}$ are the coefficients that can be obtained by calculating the overlap integral, which is given as

(10) $$A_{n,l}=\langle LG_{l,m}^{\sigma }\left(r,\phi \right)|IG_{p,m}^{\sigma }\left(\zeta ,\eta \right)\rangle$$

(11) $$B_{a,b}=\langle HG_{n_{x},n_{y}}\left(x,y\right)|IG_{p,m}^{\sigma }\left(\zeta ,\eta \right)\rangle$$

such that the value of coefficients $A_{n,l}$ and $B_{a,b}$ will always be unique for a given IG beam and will follow the condition given in Equation (12).

(12) $$\sum_{n,l}A_{n,l}^{2}=\sum_{a,b}B_{a,b}^{2}=1$$

Hence, any one beam that forms a complete set in a given coordinate system can be transformed into another complete beam defined in another coordinate system by using the transformation matrix [10], whose elements can be calculated by using the overlap integral defined in Equations (10) and (11).

#### 2.1.2. Azocarbazole Polymer

Azocarbazole is a photoswitchable chemical compound which exhibits high efficiency in optical data storage. The cis-trans photoisomerization of azocarbazole polymer makes it a suitable polymeric material for hologram recording [23,24]. When this sample is exposed to a light pattern with alternate bright and dark regions, the molecules in brighter regions undergo isomerization and relax in a particular orientation once the light is turned off. The direction of the molecular orientation depends on the polarization state of the input beam, resulting in photoinduced birefringence. The molecules in the darker region are unaffected and stay randomly oriented. Therefore, a polarized beam experiences a difference in refractive index between the bright (exposed) and dark (unexposed) areas when passing through the sample. This difference in refractive index corresponds to the recorded hologram pattern. The recorded pattern remains intact until the polymer is heated or strongly radiated. The polarization of input (writing) beams make this azocarbazole polymer anisotropic and, hence, optically birefringent. More details on the performance of the material for hologram recording and reconstruction can be found in Kinashi et al. [24].

### 2.2. Methods

#### 2.2.1. Calculation of Computer-Generated Hologram(CGH)

**Figure 4:**
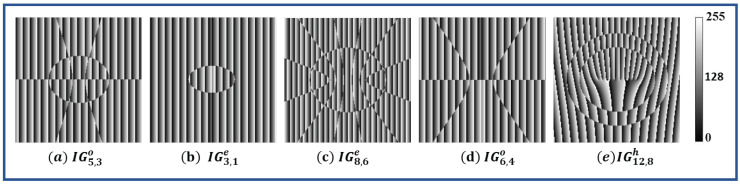
Computer-generated hologram for different Ince–Gaussian beams. (**a**) $IG_{5,3}^{o}$, (**b**) $IG_{3,1}^{e}$, (**c**) $IG_{8,6}^{e}$, (**d**) $IG_{6,4}^{o}$, (**e**) $IG_{12,8}^{h}$ for ϵ=2.

The computer-generated hologram of IG beam is calculated using the scripting language Python. In order to compute CGH, we need to generate the phase profile of IG beam of interest. For calculating phase profile, we first define a meshgrid of size n×n cm in cartesian coordinates which is divided into N×N number of pixels. We note that a rectangular aperture is usually used to simplify the plots, though Ince–Gaussian beams are elliptically symmetrical and correspond to an elliptical aperture. Using the values of rectangular aperture defined in cartesian coordinates, an elliptical coordinate (ζ,η) is defined for a fixed value of elliptical parameter (ϵ) [25]. After defining the elliptical coordinate, we divide the simulation into two parts: (i) odd Ince beam and (ii) even Ince beam. Each part is further divided into two sections: (i) odd indices (e.g., $IG_{3,1}^{\sigma }$) and (ii) even indices (e.g., $IG_{4,2}^{\sigma }$). In each section we generate the Ince polynomial by computing the coefficients in the summation and normalization constant [10]. After generating the Ince polynomial, we set various parameters such as beam waist, propagation distance, wavelength of beam, etc. Using these parameters, odd and even IG beams are generated, as defined by Equations (5) and (6). Helical Ince–Gaussian beam can easily be computed by adding even and odd IG beams. In order to compute the hologram of different IG beams, we can rewrite Equations (5), (6) and (8) in simplified form, as

(13) $$IG\left(\zeta ,\eta \right)=A\left(x,y\right)e^{i\phi (x,y)}$$

where A(x,y) is the amplitude and ϕ(x,y) is the phase of the IG beam in cartesian coordinates. A linear phase grating ($e^{i\frac{2\pi x}{d}}$) is multiplied with IG beam which serves as a carrier, where d is the period of grating. The hologram is computed by adding the phases of the IG beam and carrier beam, assuming its amplitude to be uniform across the beam cross section. Therefore, what we are generating is known as a ‘phase hologram’ and should not be confused with ‘phase-only’ hologram that is generated by iterative algorithms such as Gerchberg—Saxton (GS). The holograms of different IG beams are simulated for a meshgrid dimension of 1 cm × 1 cm with pixels values of 1200 × 1200. The elliptical parameter (ϵ) is 2.0 and propagation distance is *z* = 0 m. The wavelength of the beam is 532 nm and beam waist is 3 mm; hence, the calculated value of f(*z*) at *z* = 0 m is given as f(0) = 0.003 m. The hologram is generated for a carrier frequency of 10 pixels per cycle and is shown in Figure 4. These computer-generated holograms will be printed on azocarbazole polymers using digital hologram printing.

Commented Python3 source files for calculating phase, intensity and hologram of the different Ince–Gaussian beams and their superposition are provided as a Supplementary Materials. They include two files: (i) 〈IGbeam.py〉—the core module, which includes the necessary function to generate IG beams (this file needs no modification), and (ii) 〈example.py〉—the file where user sets the parameters and then executes it to obtain the results. The provided ‘example.py’ is pre-set to generate amplitude and phase patterns shown in Figure 1d and the corresponding hologram pattern shown in Figure 4c, when executed without any modification. To change to a different pattern or to generate the superposition states, the user may follow the comments inlined in the source code.

#### 2.2.2. Sample Synthesis

**Figure 5:**
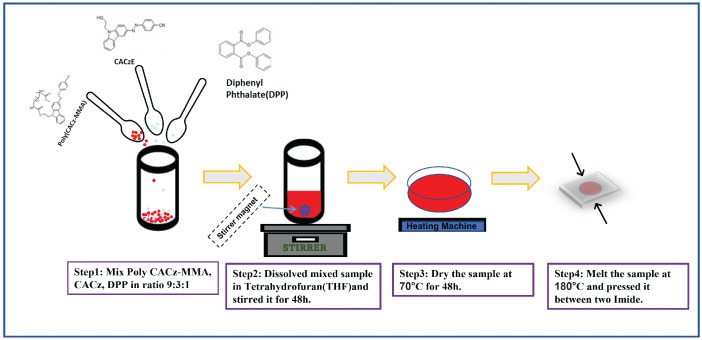
Process involved in the synthesis of azocarbazole polymer film.

**Figure 6:**
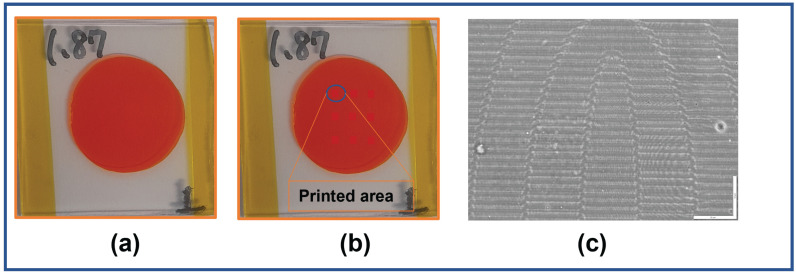
(**a**) Photograph of the synthesized azocarbazole polymer film sample. (**b**) Photograph of the sample with 9 holograms printed on film. (**c**) Phase contrast image of the printed hologram (scalebar is 50 μm in length).

The azocarbazole polymer film is prepared using a four-step process. In the first step, poly(CACzE-MMA), which is composed of 3-((4-cynophenyl)azo)-9H-carbazole-9-ethanol (CACzE) and methyl metacrylate(MMA), is mixed with CACzE and Diphenyl Phthalate(DPP) in ratio of 9:3:1 by weight. In the second step, we dissolve the above prepared sample into Tetrahydrofuran(THF) and stir for 48 h. The third step involves drying the sample at 70 ${}^{\circ }$C for another 48 h. In the last step, the sample is melted at 180–185 ${}^{\circ }$C and pressed between glass slides, where the thickness is controlled by placing polyimide spacer between the slides. The method of synthesis of azocarbazole polymer film is summarized in Figure 5. The synthesized azocarbazole polymer film is shown in Figure 6a. The thickness of the sample ranges from 30 μm to 35 μm.The sample shown in Figure 6a has thickness of 32 μm and the total thickness of sample is 1.87 mm (including thickness of glass slides).

#### 2.2.3. Digital Hologram Printing (DHP)

**Figure 7:**
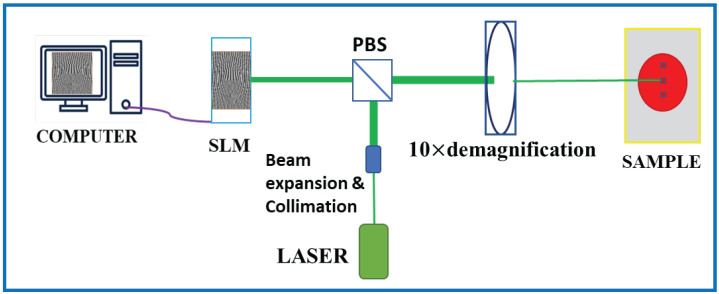
Schematic of optical setup for digital hologram printing on azocarbazole polymer film.

Digital hologram printing (DHP) is a technique used to print digitally calculated holograms (such as CGH) on a material medium using an optical setup (e.g., fringe printers) [26]. A simple schematic of the hologram printing setup which is used to print the CGH computed in Section 2.2.1 on the azocarbazole polymer film synthesized in Section 2.2.2 is shown in Figure 7. In this setup, the CGH is displayed on the spatial light modulator (SLM), where the physical size of hologram is 1 cm × 1 cm. The SLM being employed is the LC-R 1080 reflective LCOS SLM from HOLOEYE Photonics AG (HOLOEYE Photonics AG, Berlin, Germany), which has a resolution of 1920 × 1200 pixels, a pixel pitch of 8.1 μm and a frame rate of 60 Hz. A collimated beam coming out from the laser is split by a polarizing beam splitter (PBS) and the reflected beam illuminates the SLM. The phase hologram (phase modulation) pattern displayed on the SLM (phase only) is converted to amplitude modulation when the reflected beam from SLM passes through the PBS. A 10× demagnifying lens arrangement transfers the intensity pattern passing through the PBS onto the sample as refractive index modulation through a process called photoinduced birefringence. The sample is mounted on a high-precision 3-axis motion stage for accurate position of the sample w.r.t the illuminating beam (for more details about the printing setup, one may refer to Jackin et al. [19]). The laser power at the sample plane is measured to be 32 mW/mm${}^{2}$ and the recording is performed with a 5 s exposure. Multiple holograms are printed on different areas on a sample of size 3 cm × 3 cm and a photograph of the printed sample is shown in Figure 6b. The printed area of one hologram on the sample is 1 mm${}^{2}$. The diameter of the whole sample is measured to be 2 cm and we can print upto 36 holograms (avoiding the overlap between two holograms) on one sample. The sample shown in Figure 6b has a diameter of 1.87 cm and 9 holograms were printed on it. Figure 6c shows the phase contrast optical microscopic image of one of the printed holograms on azocarbazole polymer film. The refractive index changes are clearly visible as bright and dark areas in the sample.

## 3. Results

**Figure 8:**
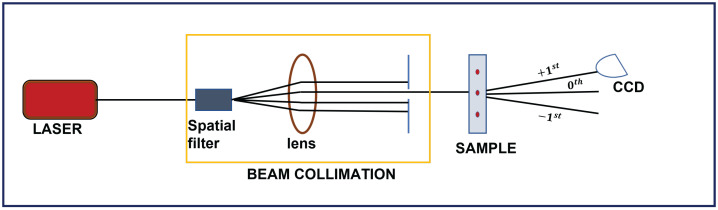
Optical setup for reconstruction of printed hologram on azocarbazole polymer film.

**Figure 9:**
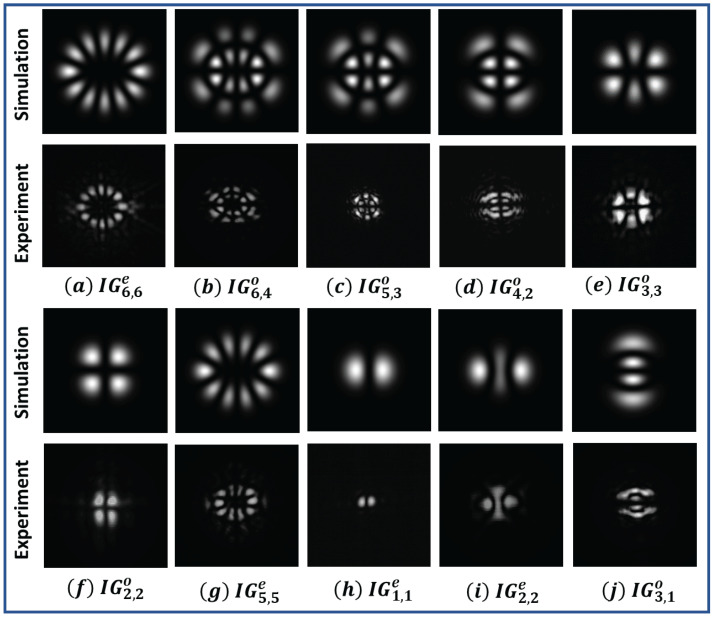
Intensity patterns of simulated and experimentally generated Ince–Gaussian beams (**a**) $IG_{6,6}^{e}$, (**b**) $IG_{6,4}^{o}$, (**c**) $IG_{5,3}^{o}$, (**d**) $IG_{4,2}^{o}$, (**e**) $IG_{3,3}^{o}$, (**f**) $IG_{2,2}^{o}$, (**g**) $IG_{5,5}^{e}$, (**h**) $IG_{1,1}^{e}$, (**i**) $IG_{2,2}^{e}$, (**j**) $IG_{5,5}^{o}$ for ϵ=2.

The printed hologram can be reconstructed using the simple setup shown in Figure 8. A collimated beam from a laser of wavelength 640 nm was passed through the printed area of the sample and the intensity pattern of the exiting radiation was observed using a CCD. The reconstructed intensity pattern for the different odd IG beams ($IG_{p,m}^{o}$) and even IG beams ($IG_{p,m}^{e}$) are shown in Figure 9a–j. The experimentally observed intensity patterns were compared with the simulated intensity patterns and were found to be in good agreement.

**Figure 10:**
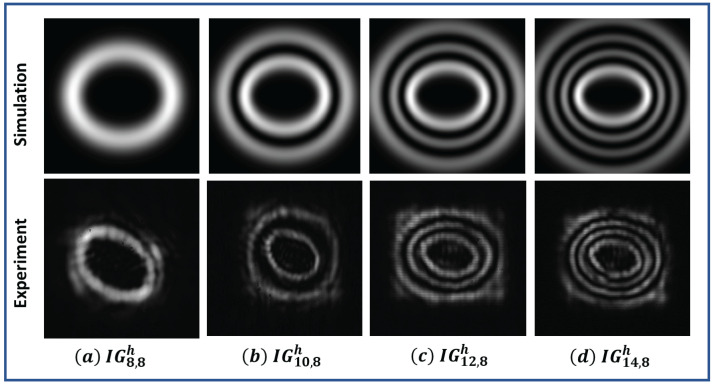
Intensity pattern of simulated and experimentally generated helical Ince–Gaussian beams (**a**) $IG_{8,8}^{h}$, (**b**) $IG_{10,8}^{h}$, (**c**) $IG_{12,8}^{h}$, (**d**) $IG_{14,8}^{h}$ for ϵ=2.

The ring-type helical Ince–Gaussian beams ($IG_{p,m}^{h}$) were also generated using the same setup. The experimental verification of generated intensity profiles for different helical Ince–Gaussian beams are shown in Figure 10.

The diffraction efficiency (η(%)) for the generated IG beams was calculated using Equation (14), given as:

(14) $$\eta \left(\%\right)=\frac{I_{+1}}{I_{t}}\times 100$$

where $I_{+1}$ is the intensity of +1 order of the diffracted beam and $I_{t}$ is the total transmitted intensity, which can be measured by passing a collimated beam through the unprinted sample. The calculated value of diffraction efficiency is 30%. This is in close agreement with the theoretical maximum efficiency of the Raman–Nath regime, which is 33.9%. Hence, we can conclude that the efficiency obtained from our sample is very high. The retention time of the printed hologram on the sample is nearly 50 days, which makes the azocarbazole polymer suitable for writing once and later reading multiple times (permanent storage). However, with the application of heat on purpose, the written pattern can be erased, and the sample be reused for another recording (unlike photopolymers, which are non-reusable).

**Figure 11:**
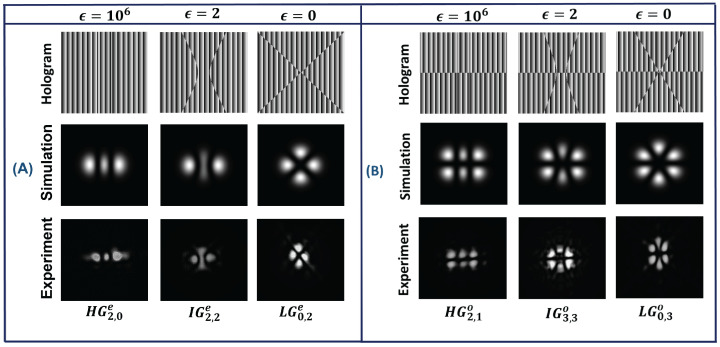
(**A**) Conversion of $IG_{2,2}^{e}$ beam (second column) into $HG_{2,0}^{e}$ beam (first column) and $LG_{0,2}^{e}$ beam (third column) for different ϵ values. (**B**) Conversion of $IG_{3,3}^{o}$ beam (second column) into $HG_{2,1}^{o}$ beam (first column) and $LG_{0,3}^{o}$ beam (third column) for different ϵ values.

IG beams were transformed into other structural beams by changing their elliptical parameter (ϵ) values. Initially, an even IG beam ($IG_{2,2}^{e}$) and an odd IG beam ($IG_{3,3}^{o}$) were generated for ϵ = 2 and then, by changing the value of ϵ to $10^{6}$, the $IG_{2,2}^{e}$ beam changes to $HG_{2,0}^{e}$ and the $IG_{3,3}^{o}$ beam changes to $HG_{2,1}^{e}$. For ϵ = 0, the $IG_{2,2}^{e}$ beam changes to $LG_{0,2}^{e}$ and $IG_{3,3}^{o}$ changes to $LG_{0,3}^{e}$. The corresponding generated beams are shown in Figure 11.

**Figure 12:**
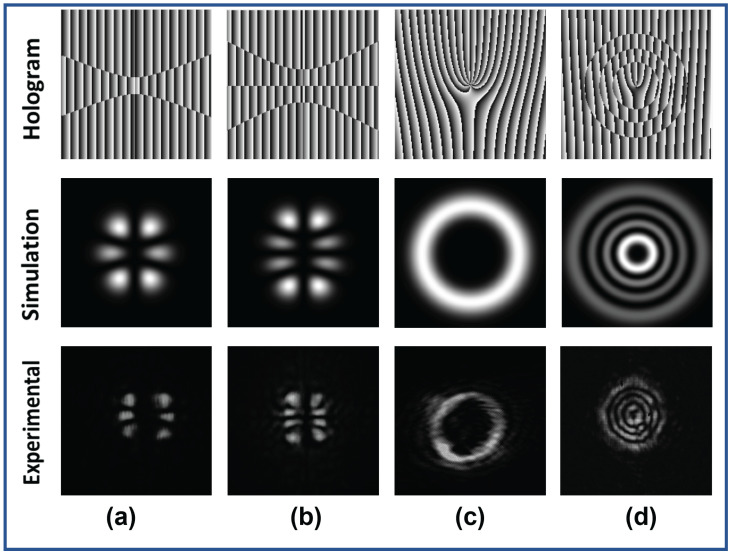
Holograms (first row) of superposition of different IG beams. Simulation (second row) and experimental (Third Row) intensity profile of superposition of IG beams (**a**) $IG_{3,3}^{e}$+$IG_{3,1}^{e}$ for ϵ=2, (**b**) $IG_{4,4}^{o}$+$IG_{4,2}^{o}$ for ϵ=2, (**c**) $IG_{9,9}^{o}$+$IG_{9,9}^{e}$ for ϵ=0, (**d**) $IG_{9,3}^{o}$+$IG_{9,3}^{e}$ for ϵ=0.

The superposition states of IG beams were also generated, which is important in the domain of optical communication. We generated the superposition of an IG beam for ϵ=2 and ϵ=0 and they are shown in Figure 12. Figure 12a shows the superposition of $IG_{3,1}^{e}$ and $IG_{3,3}^{e}$, while Figure 12b shows the superposition of $IG_{4,4}^{o}$ and $IG_{4,2}^{o}$, for ϵ=2. The superposition of $IG_{9,9}^{o}$ and $IG_{9,9}^{e}$ and the superposition of $IG_{9,3}^{o}$ and $IG_{9,3}^{e}$ for ϵ=0 is shown in Figure 12c,d, respectively. Figure 12c,d corresponds to the generation of a vortex and a vortex with circular rings, respectively. Information can be encoded into these superposition states and can be transmitted to the receiver who can use the orthogonality relation between these beams to decode the information. On the other hand, plane electromagnetic beams do not possess orthogonality and hence are difficult to separate once they are superposed (unless angular, polarization and wavelength multiplexing, etc., are employed). Moreover, certain modes of IG beams also possess orbital angular momentum (vortex modes), which can be used to encode information directly in terms of topological charges.

The orthonormality constant was calculated by evaluating Equation (7) numerically in Python. The process involved in the calculation of an orthonormality constant consists of four steps. In the first step, the dot product between two simulated IG beams was calculated; then, in the second step, the total number of pixels occupied by this dot product in the region defined in the meshgrid was calculated. In the third step, the area occupied by 1 pixel was calculated by using formula ${\left(\frac{L}{N-1}\right)}^{2}$, such that L is the length of the meshgrid and N is the total number of pixels. Finally, in the last step, the orthonormality constant was calculated by multiplying the total number of pixels occupied by the dot product with the area of one pixel.

**Figure 13:**
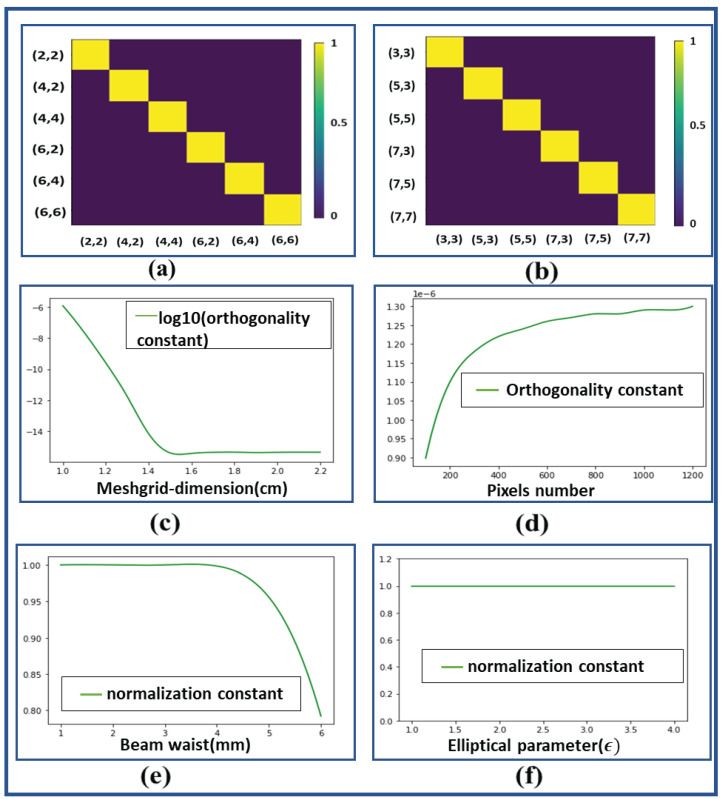
(**a**) Orthogonality relation between Ince–Gaussian beam of even indices for ϵ = 2. (**b**) Orthogonality relation between Ince–Gaussian beam of odd indices for ϵ = 2. (**c**) Variation ins log(orthogonality constant) for beams $IG_{5,3}^{e}$ and $IG_{3,1}^{e}$ with meshgrid size (L) for ϵ = 2. (**d**) Variation in orthogonality constant for beams $IG_{5,3}^{e}$ and $IG_{3,1}^{e}$ with pixels number (N) for ϵ = 2. (**e**) Variation in normalization constant for beams $IG_{4,2}^{o}$ and $IG_{4,2}^{o}$ with beam waist ($W_{0}$) for ϵ = 2. (**f**) Variation in normalization constant for beams $IG_{4,4}^{o}$ and $IG_{4,4}^{o}$ with elliptical parameter (ϵ).

The investigated orthonormality relation for different configurations is shown in Figure 13. Figure 13a,b shows the orthogonality relation between even and odd indices. The variation in orthogonality relation with different parameters such as beam waist ($W_{o}$), meshgrid dimension (n × n), pixels numbers (N × N) and elliptical parameter (ϵ) was analysed and the results thus obtained are shown in Figure 13c–f. The logarithmic variation in orthogonality constant with meshgrid sizes for modes $IG_{5,3}^{e}$ and $IG_{3,1}^{e}$ for ϵ=2 can be seen from Figure 13c for a given beam waist of 3 mm and pixel number of 512 × 512. We found that for a meshgrid size greater than 1.3 cm × 1.3 cm, the orthogonality relation is mostly satisfied. Figure 13d shows the variation in orthogonality constant between modes $IG_{5,3}^{e}$ and $IG_{3,1}^{e}$ for ϵ=2 with the pixel number for a meshgrid size of 1 cm × 1 cm and a beam waist of 3 mm. We find that the orthogonality relation in this case deteriorates when we increase the pixel number. Figure 13e manifests the variation in the normalization constant (factor of 1) between modes $IG_{4,2}^{o}$ and $IG_{4,2}^{o}$ for ϵ=2 with a varying beam waist for a meshgrid size of 1 cm × 1 cm and pixel number of 1000 × 1000. Here, we find that, for beam waist size less than 4 mm, the normalization relation is well satisfied and after that, the normalization relation starts violating the ideal case. Figure 13f conveys the variation in normalization constant with elliptical parameter between modes $IG_{4,4}^{o}$ and $IG_{4,4}^{o}$ for a meshgrid size of 1 cm × 1 cm, pixels number 1000 × 1000 and beam waist 3 mm. We observe that the normalization constant is well-defined at all values of elliptical parameters, which indicates the validation of the orthonormality relation even when the IG beam changes to an HG or LG beam. Hence, from all these observations, we can conclude that a perfect combination of meshgrid dimension, pixel numbers and beam waist is important for generating IG beams and their superposed state to access their application in optical communication.

## 4. Conclusions

A combination of digital hologram printing and an azocarbazole polymer was used to overcome the high cost, large footprint and non-scalablity of existing IG beam generators. Intensity, phase and the corresponding CGH of different IG beams were computed using the open source Python programming language. The simulated and experimentally generated intensity profiles of IG beams were found to be in good agreement, which determines that the beams were generated successfully. The conversion of an IG beam into other structural beams, such as LG and HG, is also demonstrated by simulation and was verified experimentally. In order to access the applicability of IG beams in optical communication, the superposed states of different IG and LG beams were generated and their orthogonality relations were examined. The reported compact and scalable IG-beam-generation technique could open up new possibilities in structural-beam science. Further improvement to this technique can be made by increasing the printing area of a hologram on polymer film, which will possibly increase the diffraction efficiency and assist larger mode generation. Other structural beams defined in different coordinates, such as Bessel–Gaussian beams [27], Mathieu–Gauss beams [28], Airy–Gauss beams [29], hypergeometric Gaussian beams [30], etc., can also be generated using the proposed technique.

## Data Availability

Not applicable.

## Supplementary Materials

Supporting python files are available at https://www.mdpi.com/article/10.3390/jimaging8050144/s1.

## Abbreviations

The following abbreviations are used in this manuscript:

IG	Ince–Gaussian
HG	Hermite Gaussian
LG	Laguerre Gaussian
CGH	Computer generated hologram
DHP	Digital Hologram printing
CCD	Charge coupled device
SLM	Spatial light modulator
MMA	Methyl metacrylate
DPP	Diphenyl pthalate
THF	Tetrahydrofuran
PBS	Polarizing beam splitter
